# Quantitative acoustic differentiation of cryptic species illustrated with King and Clapper rails

**DOI:** 10.1002/ece3.4711

**Published:** 2018-11-20

**Authors:** Lydia L. Stiffler, Katie M. Schroeder, James T. Anderson, Susan B. McRae, Todd E. Katzner

**Affiliations:** ^1^ Division of Forestry and Natural Resources West Virginia University Morgantown West Virginia; ^2^ Department of Biology East Carolina University Greenville North Carolina; ^3^ U.S. Geological Survey Forest & Rangeland Ecosystem Science Center Boise Idaho; ^4^ Present address: Warnell School of Forestry and Natural Resources University of Georgia Athens Georgia; ^5^ Present address: Department of Biology University of Massachusetts Amherst Amherst Massachusetts

**Keywords:** acoustic differentiation, nonparametric, parametric, Rallidae, *Rallus crepitans*, *Rallus elegans*

## Abstract

Reliable species identification is vital for survey and monitoring programs. Recently, the development of digital technology for recording and analyzing vocalizations has assisted in acoustic surveying for cryptic, rare, or elusive species. However, the quantitative tools that exist for species differentiation are still being refined. Using vocalizations recorded in the course of ecological studies of a King Rail (*Rallus elegans*) and a Clapper Rail (*Rallus crepitans*) population, we assessed the accuracy and effectiveness of three parametric (logistic regression, discriminant function analysis, quadratic discriminant function analysis) and six nonparametric (support vector machine, CART, Random Forest, *k*‐nearest neighbor, weighted *k*‐nearest neighbor, and neural networks) statistical classification methods for differentiating these species by their *kek* mating call. We identified 480 *kek* notes of each species and quantitatively characterized them with five standardized acoustic parameters. Overall, nonparametric classification methods outperformed parametric classification methods for species differentiation (nonparametric tools were between 57% and 81% accurate, parametric tools were between 57% and 60% accurate). Of the nine classification methods, Random Forest was the most accurate and precise, resulting in 81.1% correct classification of *kek* notes to species. This suggests that the mating calls of these sister species are likely difficult for human observers to tell apart. However, it also implies that appropriate statistical tools may allow reasonable species‐level classification accuracy of recorded calls and provide an alternative to species classification where other capture‐ or genotype‐based survey techniques are not possible.

## 1 INTRODUCTION

Reliable species identification is vital for survey and monitoring programs. This task can be challenging when faced with cryptic, rare, or elusive species (Bickford et al., 2007; Thompson, 2004). For taxa that are difficult to locate, trap, and monitor visually, conservation biologists often rely upon acoustic surveying of vocalizations to identify species and individuals (Conway, 2011; Oswald, Rankin, Barlow, & Lammers, 2007; Parsons & Jones, 2000; Steiner, 1981; Stiffler, Anderson, & Katzner, 2018a ).

Vocalizations can be identified qualitatively by experienced surveyors or quantitatively by using characteristics of spectrograms to compare recorded calls or unknown origin to known‐individual recordings (Conway, 2011; Parsons & Jones, 2000; Steiner, 1981). However for sister species that are difficult to distinguish, qualitative differentiation methods may be too subjective and quantified sound characteristics based on spectrograms are recommended for reliable species identification (Lambert & McDonald, 2014; Russo & Voigt, 2016). The digital technology for recording and analyzing vocalizations has recently been developed, and the quantitative tools that exist for species differentiation are still being refined.

The most commonly used tools for quantitative acoustic differentiation between species are parametric models such as discriminant function analysis or logistic regression (Biscardi, Orprecio, Fenton, Tsoar, & Ratcliffe, 2004; Oswald et al., 2007; Smith, Newman, Hoffman, & Fetterly, 1982; Steiner, 1981; Teixeira & Jesus, 2009; Vaughan, Jones, & Harris, 1997). Newer nonparametric models such as *k*‐nearest neighbor and neural networks have also been successful at species classification (Britzke, Duchamp, Murray, Swihhart, & Robbins, 2011; Parsons, 2001; Redgwell, Szewczak, Jones, & Parsons, 2009). Comparisons between parametric and nonparametric approaches suggest that no singular approach works best under all circumstances. For example, in some cases neural networks outperform all other classification methods (Britzke et al., 2011; Parsons & Jones, 2000; Redgwell et al., 2009). However, in one study of vocalizations of 20 species of bats, multiple discriminant analysis performed better than neural networks (Preatoni et al., 2005).

**Figure 1:**
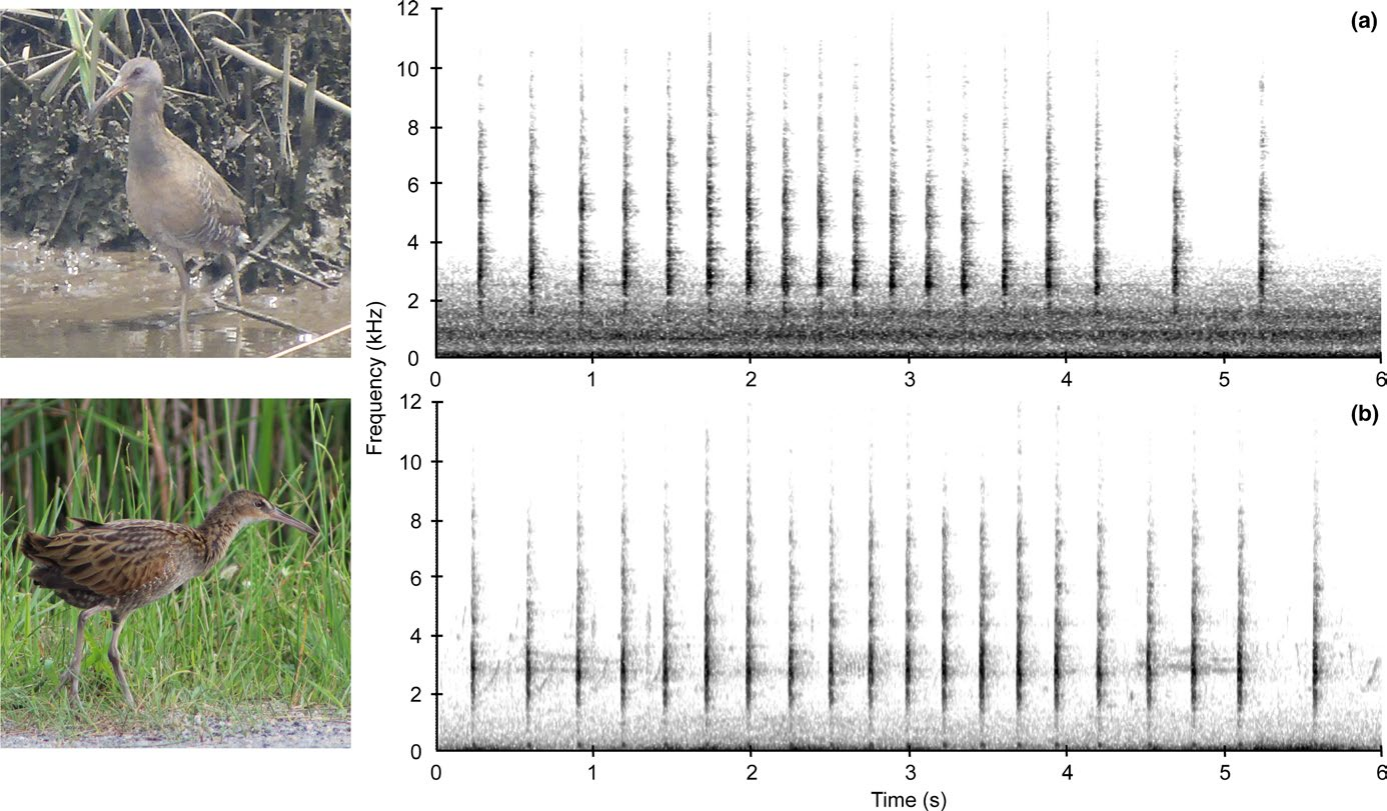
Morphological and spectrographic comparison of a Clapper Rail (a) and a King Rail (b). Spectrograms depict a six second *kek* call segment from an individual of each species

Although several species of secretive marsh birds produce qualitatively distinguishable calls, others do not. For example, the calls produced by the Clapper Rail (*Rallus crepitans*) and its sister species the King Rail (*Rallus elegans*) are similar in structure and variable among individuals, making qualitative aural differentiation difficult (Conway, 2011; Graves, 2001; Zembal & Massey, 1987; Figure 1). Identifying these species accurately in the field is important because both have experienced significant population declines due to wetland loss and fragmentation (Correll et al., 2017). Furthermore, although the two species are similar, their conservation status and suggested management practices differ, and thus, it is important to find mechanisms to effectively and accurately identify and survey for these species (Cooper, 2008; Kushlan et al., 2006).

The overarching goal of this study was to examine quantitative statistical tools to distinguish vocalizations of King and Clapper rails collected during surveys. To do this, a collaborative joint effort was established between researchers at West Virginia University and East Carolina University. Each university was conducting independent projects on rail vocalizations with different overarching project goals. Researchers at West Virginia University investigated rail distributions along the Pamunkey and Mattaponi Rivers, Virginia, USA using acoustic surveying techniques (Stiffler et al., 2018a, 2018b, 2017), while researchers at East Carolina University explored the vocal behaviors of King rails in a long‐term monitored population in North Carolina, USA (Schroeder, 2018). We used recordings of known King and Clapper rail *kek* vocalizations and quantitatively characterized them with seven standardized acoustic parameters. We then compared the accuracy and effectiveness of three parametric and six nonparametric statistical classification tools to (a) determine whether these two species could reliably be differentiated acoustically, and (b) identify which method performed best at differentiating the two species.

## 2 METHODS

### 2.1 Study species

King and Clapper rails coexist along a salinity gradient within marshes of the Atlantic and Gulf coasts of the United States. King rails inhabit both freshwater and brackish marshes, while Clapper rails reside in tidal salt marshes (Meanley, 1985). However, both species are sympatric in transitional zones of intermediate brackish‐salt marshes (Meanley, 1969; Meanley & Wetherbee, 1962), and, where they coexist, they may hybridize (Chan, Hill, Maldonado, & Fleischer, 2006; Meanley & Wetherbee, 1962). The two species can be differentiated based on subtle variations in morphology (i.e., size, plumage), physiology (i.e., osmoregulation by salt glands), and genetics (i.e., mitochondrial and nuclear DNA) (Chan et al., 2006; Conway, Hughes, & Moldenhauer, 1988; Eddleman & Conway, 1994; Maley & Brumfield, 2013; Olson, 1997; Reid, Meanley, & Fredrickson, 1994).

King and Clapper rails produce eight distinct calls using variants of a single note (Massey & Zembal, 1987; Meanley, 1969). One of their most frequent calls is the *kek*, which consists of a single note repeated multiple times. Intraspecific and within‐individual variation occurs with *kek* note structure and calling rates in response to external stimuli (Massey & Zembal, 1987; L. L. Stiffler, & K. M. Schroeder, personal observations). During the breeding season, unpaired males use the repeated *kek* call for mate advertisement as well as in territorial displays when paired (Kolts & McRae, 2017; Meanley, 1969; Zembal & Massey, 1987).

### 2.2 Field data collection

We recorded calls from King and Clapper rails at two study sites ~135 km apart. Known populations of only one of the two species inhabit each site. We did not use playback to elicit calls from either species, but instead recorded calls passively.

Clapper rails were recorded May–July 2015 within Eltham Marsh near West Point, Virginia, USA. Eltham Marsh is a ~288 ha privately owned brackish tidal marsh located at the confluence of the York and Pamunkey rivers within the Chesapeake Bay. Vegetation in lower areas was dominated by smooth cordgrass (*Spartina alterniflora*), while the higher, irregularly flooded areas were dominated by saltmeadow cordgrass (*Spartina patens*) and big cordgrass (*Spartina cynosuroides*). Recordings were taken using a Song Meter SM3 (Wildlife Acoustics, Maynard, MA, USA) at 24 kHz and 16‐bit deployed in rotation between 15 random locations within the marsh, each one at least 400 m from every other survey location (Conway, 2011), at least 50 m from marsh edge, and easily accessible by boat from the Pamunkey River. Animal capture and population genetic surveying of the marsh confirmed that Clapper rails were the only *Rallus* species found within the marsh (Coster et al., 2018; G. Costanzo and S. Harding unpublished data).

King Rail recordings were collected April–June 2016 at Mackay Island National Wildlife Refuge (NWR) in northeastern North Carolina, USA. Mackay Island is a 3,300 ha freshwater and brackish marsh centrally located on the Atlantic Flyway in the Southeast Coastal Plain. The refuge includes 550 ha of impoundments managed for overwintering waterfowl, as well as extensive natural marshes subject to prescribed burn (Rogers, Collazo, & Drew, 2013). King rails are the only species of long‐billed rail breeding at the site, and detailed study of King Rail ecology and behavior has been ongoing at Mackay Island NWR for the last 7 years (for further information about the study population, see Clauser & McRae, 2016; Clauser & McRae, 2017; Kolts & McRae, 2017). Recordings were made using a Song Meter SM4 (Wildlife Acoustics) at 44.1 kHz and 16 bit. Two SM4s were deployed in rotation among 10 different locations on the refuge. Locations were no <400 m apart (Conway, 2011) and were selected based on auditory and visual confirmation of King Rail presence. Additional recordings were made opportunistically in different locations using a handheld linear pulse‐code modulation (PCM) recorder (Sony, New York, NY, USA) and ME 66 shotgun microphone (Sennheiser, Old Lyme, CT, USA; 44.1 kHz, 16‐bit).

The selection of field sites and seasonal timing of our surveys makes it unlikely we would encounter hybrids within our systems. Ecological segregation occurs between the King and Clapper rail on the basis of habitat salinity (Maley & Brumfield, 2013). In our genetic and trapping surveys over several years, we found that the brackish Eltham marsh contained exclusively Clapper rails, while the freshwater marshes of Mackay Island NWR are inhabited exclusively by King rails. Although King rails can be found in saltmarshes during migration stopovers, they leave these areas prior to breeding (Meanley, 1969; Reid et al., 1994). Thus, by surveying during the breeding season, we have limited the potential for misidentifying recordings of vocalizations.

### 2.3 Processing and preparation of acoustic data

We visualized all calls using Raven Pro software (Bioacoustics Research Program, 2014). We selected for analysis 480 King Rail and 480 Clapper Rail *kek* notes. Recordings were selected between the hours of 0630–0830 and 1800–2015 Eastern Daylight Time (EDT) to account for traditional marsh bird monitoring protocols during sunrise and sunset. During these time frames, difference between morning and evening call patterns and structure were marginal (Schroeder, 2018; Stiffler et al., 2017). We chose to use single *kek* notes instead of *kek* calls (a series of *kek* notes in sequence) since call length was often difficult to ascertain, and bouts of calling sometimes continue for hours with periodic pauses (Massey & Zembal, 1987). Notes were selected that did not overlap calls of other wetland species such as Red‐winged Blackbirds (*Agelaius phoeniceus*), Marsh Wrens (*Cistothorus palustris*), and Killdeer (*Charadrius vociferus*). All *kek* notes from both species were truncated to only include frequencies between 1.5–5 kHz. This allowed us to exclude prominent low frequency background noises in recordings taken from Eltham Marsh and cricket calls at around 5.5 kHz in Mackay Island NWR recordings. In spite of this truncation, all *kek* note selections captured the major harmonic (Massey & Zembal, 1987).

**Figure 2:**
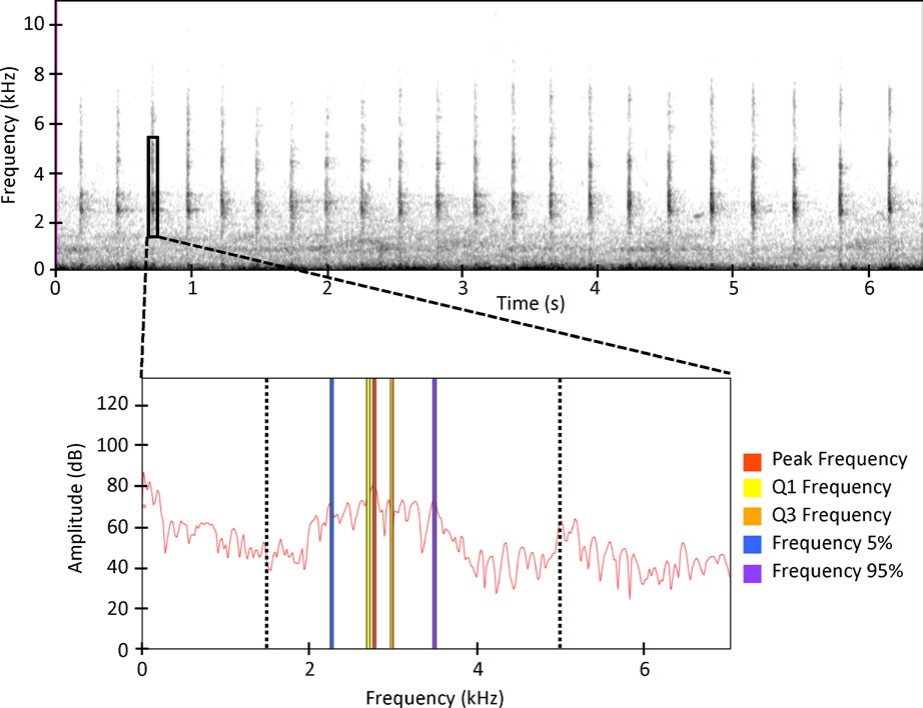
Digitization and example measurement of a typical Clapper Rail *kek* call in Raven Pro. Notes were selected from the spectrogram then measured in the power spectrum. Parameters defined in Table S1. To minimize underlying background noise, only regions between 1,500 and 5,000 Hz were included in parameter measurement, as represented by the dotted black lines

We measured and quantified the following seven parameters from each *kek* note: (a) Peak Frequency, (b) First Quartile Frequency, (c) Third Quartile Frequency, (d) Inter‐quartile Range (IQR) Bandwidth, (e) Frequency 5%, (f) Frequency 95%, and (g) Bandwidth 90% (derived from Charif, Waack, & Strickman, 2010; each variable is described in Supporting Information Table S1; Figure 2). Since we truncated each call note to the portion between 1.5 and 5 kHz, we excluded from the list of parameters we considered, the Minimum and Maximum Frequencies. Although Pulse Rate and Duration were originally considered for analysis, high variation in these parameters within and between individuals of the same species meant that they had limited predictive power for species classification. Parameters were measured from the power spectrum (Hann window, window size 1,024 samples). To account for different sampling frequencies during recording, a Discrete Fourier Transformation (DFT) of 2,048 samples was used for King Rail calls and 1,024 samples for Clapper rails. This resulted in frequency resolutions of 21.5 and 23.4 Hz for King and Clapper rails, respectively.

### 2.4 Statistical analysis

We first evaluated Spearman's rank correlations between parameters to determine which parameters to retain and which to remove from further analyses. Removal of highly correlated parameters ensures the assumption of little to no multicollinearity exist for parametric classification tools. Of the seven parameters we considered, two (IQR Bandwidth, Bandwidth 90) were highly correlated with other parameters (*r* > 0.70) and thus removed from further consideration (Supporting Information Table S2). We retained the remaining five parameters for use in statistical analyses (variance‐inflation factor <3; Fox & Monette, 1992). We performed all statistical analyses using Program R (R Development Core Team, 2013).

The nine quantitative classification methods we used for species differentiation were as follows: logistic regression, support vector machine, classification and regression tree (CART), Random Forests, linear discriminant function analysis (DFA), quadratic DFA, *k*‐nearest neighbor, weighted *k*‐nearest neighbor, and neural networks. Each is described in detail below. Using such a broad range of techniques, we allow for a variety of model development approaches. We randomly assigned 70% of the *kek* notes to the model building dataset and we reserved the remaining 30% for model cross‐validation. The model building dataset served to train the classification functions.

For each approach, we calculated accuracy, precision, sensitivity, specificity, area under the curve (AUC), and Cohen's kappa coefficient (Landis & Koch, 1977; Sokolova & Lapalme, 2009). Accuracy is a measure of the model's ability to correctly assign individual *kek* notes to their proper species. We calculated overall classification accuracy rates for each model using confusion matrices. Since our models were assigning calls to one of two species, interpreting the accuracy of a given analysis must be performed relative to the accuracy expected by chance alone (i.e., 50%). Precision represents the class agreement of the data for Clapper rails given by the model. Sensitivity represents a model's effectiveness in classifying Clapper rails, while specificity represents a model's effectiveness in classifying King rails. The area under the curve (AUC) describes the model's ability to avoid false species’ identifications. We used Cohen's kappa coefficients (*Κ*) to evaluate the chance‐adjusted classification agreement between the true classification and the model‐predicted classification (Landis & Koch, 1977; Viera & Garrett, 2005). Kappa is a metric standardized between −1 and 1, where 1 is perfect agreement and 0 is agreement by chance alone (Landis & Koch, 1977). We conducted 1,000 iterations of model building and cross‐validation to account for variability in model performance due to random assignment of *kek* notes.

The two discriminant function analyses and logistic regression are all parametric approaches to classification. Linear discriminant function analysis classifies *kek* notes to groups based on orthogonal linear functions derived from the five parameters by maximizing the variation between species, assuming equality of covariance matrix among species (Venables & Ripley, 2002). Quadratic discriminant function analysis relaxes the assumption of a single covariance matrix for both species by estimating separate covariance matrices using quadratic functions (Venables & Ripley, 2002). Both discriminant function analyses were performed using the R package “MASS” (Ripley et al., 2018). Logistic regression classifies individuals into species by estimating probabilities conditional to the five parameters using a logistic function (Press & Wilson, 1978).

Neural network classification, CART, Random Forests, support vector machines, *k*‐nearest neighbor, and weighted *k*‐nearest neighbor are nonparametric methods that assume no distribution for model development. *K*‐nearest neighbor assigns species classification for an individual note based on the majority of species’ identities of the note's *k‐*nearest neighbors (Hechenbichler & Schliep, 2004; Venables & Ripley, 2002). We used the R package “class” for *k*‐nearest neighbor classification and the R package “kknn” for weighted *k*‐nearest neighbor classification (Ripley & Venables, 2015; Schlierp, Hechenbichler, & Lizee, 2016). We evaluated the performances of *k* ranging from 0 to 20 and selected *k* = 1 for analysis because it resulted in the largest reduction in classification error. Weighted *k*‐nearest neighbor performs similarly, but weights the influence of the neighbors by distance, whereby closer neighbors provide higher weights for species classification (Hechenbichler & Schliep, 2004). We evaluated the performances of *k* ranging from 0 to 20 and selected *k* = 5 for analysis because it resulted in the largest reduction in classification error.

The CART decision tree recursively partitions the data into two groups using a splitting rule to identify the split to use at each node (Steinberg & Colla, 2009). Single classification trees are grown to maximal size then pruned back until the highest predictive performance is achieved. In contrast, the Random Forest grows multiple classification trees in which each tree “votes” on the classification based on how each tree splits the data at nodes (Breiman, 2001). The forest chooses the overall classification having the most “votes” by aggregating across all trees. We used the R package “rpart” to build the CART classification trees and the R package “randomForest” to conduct our Random Forest analysis (Liaw & Wiener, 2018; Therneau, Atkinson, & Ripley, 2018).

Support vector machines rely on learning algorithms to perform discriminative classification by creating separation splines between species through iterative training (Vapnik, Golowich, & Smola, 1996). The support vector machine learns to tell the difference between the two species by optimizing the separating hyperplane that maximizes the distance between the closest *kek* notes lying on the boundaries (Bennett & Campbell, 2000). We performed this analysis using the R package “e1071” (Meyer et al., 2018).

Neural networks are algorithms that simulate the human brain through learning and memorization of mathematical relationships (Venables & Ripley, 2002). For the neural network construction, we used the R package “neuralnet” to build a feed‐forward, resilient back‐propagation classification neural network (Fritsch, Guenther, Suling, & Mueller, 2016; Riedmiller & Braun, 1993; Smith, 1996; Venables & Ripley, 2002). The input layer consisted of the five *kek* note parameters. The network output was a single neuron for species classification. We chose a structure with a single hidden layer for simplicity, but varied the number of neurons per hidden layer between 1 and 18. Neurons within the hidden layer form interaction terms based on weights of the connection between each input neuron and hidden neuron (Venables & Ripley, 2002; Warner & Misra, 1996). We trained each neural network on 1,000 repetitions prior to assessing overall accuracy. We identified the most suitable network architecture (0 hidden neurons) as the one that produced the highest accuracy rate.

## 3 RESULTS

The mean and median values of all five parameters taken from the 960 *kek* notes were similar between species (Table 1). All nonparametric classification methods performed slightly better for Clapper rails (sensitivity > specificity), while all parametric classification methods performed better for King rails (sensitivity < specificity; Table 2). Among all classification methods, accuracy and AUC provided identical assessment of model fit. Overall, each classification model performed better than expected by chance alone and in general, nonparametric techniques performed better than parametric models. Random Forest and weighted *k*‐nearest neighbor were the only two methods that resulted in *Κ* coefficients >50%. Random Forest was the most accurate and precise classification tool for individual *kek* call notes (81.1% overall), and this technique correctly classified 81.7% of Clapper Rail and 80.6% of King Rail call notes. The two *k*‐nearest neighbor approaches and the support vector machine approach were less effective in classification, providing just over 70% accuracies. Of the nonparametric approaches, CART and Neural Networks were the worst (both <62% accurate). The three parametric approaches, logistic regression and quadratic and linear DFA, classified call notes relatively poorly (57%–60% accurate; Table 2)

**Table 1:** Means, medians, standard deviations (*SD*), and coefficients of variation (C.V., as a percent) for five variables (all measured in Hz) from *kek* call notes of Clapper rails monitored in Virginia, USA during 2015 and King rails in North Carolina, USA during 2016

	Clapper rails				King rails			
	Mean	Median	*SD*	C.V.	Mean	Median	*SD*	C.V.
Peak frequency	2,824	2,742	529	18	2,808	2,756	476	17
Q1 frequency	2,482	2,508	337	13	2,556	2,562	309	12
Q3 frequency	3,284	3,258	416	12	3,254	3,112	530	16
Frequency 5%	1979	1922	280	14	2096	2,132	323	16
Frequency 95%	4,175	4,312	579	13	3,997	4,059	587	15

**Table 2:** Classification efficacy for individual King and Clapper rail *kek* call notes from North Carolina and Virginia, USA, respectively, during 2015 and 2016, classified to species and ranked according to model performance

Classification model	Class	Accuracy	Precision	Sensitivity	Specificity	AUC	*Κ*
Random Forest	N	0.811	0.809	0.817	0.806	0.811	0.623
Weighted *k*‐nearest neighbor	N	0.755	0.743	0.782	0.729	0.755	0.511
*k*‐nearest neighbor	N	0.733	0.722	0.759	0.707	0.733	0.466
Support vector machine	N	0.714	0.707	0.734	0.694	0.714	0.428
CART	N	0.617	0.602	0.702	0.531	0.617	0.233
Quadratic DFA	P	0.597	0.596	0.602	0.592	0.597	0.194
Neural network	N	0.575	0.566	0.673	0.477	0.575	0.150
Logistic regression	P	0.566	0.567	0.563	0.570	0.566	0.132
Linear DFA	P	0.566	0.567	0.562	0.570	0.566	0.132

Note

The models were logistic regression, support vector machine, classification and regression tree (CART), Random Forest, linear discriminant function analysis (DFA), quadratic DFA, *k*‐nearest neighbor, weighted *k*‐nearest neighbor, and neural networks. In all cases, mean classification rates were determined using cross‐validation (70%/30%) for 1,000 iterations. Class represents either parametric (P) or nonparametric (N) test types. Accuracy represents the overall effectiveness of a model, and Precision describes the class agreement for Clapper rails given by the model. Sensitivity represents model effectiveness in classifying Clapper rails, while specificity represents model effectiveness in classifying King rails. The area under the curve (AUC) describes the model's ability to avoid false species identifications. Cohen's kappa coefficients (*Κ*) represent the corrected classification score for inter‐rate agreement by chance.

The most important parameters for accurate classification were similar for the top four classification methods, although the percent of relative importance varied (Table 3). Frequency 5% and Q3 Frequency were the most important parameters accounting for >60% of relative importance for model classification. Q1 Frequency and Peak Frequency were less important, but still accounted for most of the remaining importance. Frequency 95% was generally unimportant to any of the top classification models.

**Table 3:** Relative importance of five acoustic parameters used in classification of *kek* notes from King and Clapper rails

Classification method	Acoustic parameter				
	Frequency 95%	Peak frequency	Q1 frequency	Q3 frequency	Frequency 5%
Random Forest	0.000	0.2516	0.3060	0.3757	0.4611
Support vector machine	0.000	0.2608	0.3090	0.3627	0.4131
*k*‐nearest neighbor	0.000	0.2491	0.3036	0.3760	0.4102
Weighted *k*‐nearest neighbor	0.000	0.2508	0.2955	0.3310	0.3965

Note

Comparison is between classification methods that resulted in >70% accuracy.

## 4 DISCUSSION

Our analyses showed that it was possible to statistically separate recorded *kek* call notes of King and Clapper rails. However, our work also demonstrated that the effectiveness with which this could be performed depended greatly on the statistical tool used for classification. As such, our findings have relevance both to surveys for conservation and management of these species, and to our broader understanding of the utility of statistical tools for classification of vocalizations based on variation in frequency parameters.

### 4.1 Classification efficacy

We found substantial variation in accuracy among the nine classification methods we tested. Random Forests and weighted *k*‐nearest neighbors were the top two performing models with *Κ* coefficients >50%, suggesting moderate to substantial agreement between true and model‐predicted classifications (Landis & Koch, 1977). Although both Random Forests and weighted *k*‐nearest neighbor methods rely upon the same information, each analysis offered differing advantages and disadvantages for classification. Random Forest combines results from multiple decision trees, thus overcoming the problem of overfitting symptomatic of CART (Breiman, 2001). As a consequence, Random Forest possesses a flexible framework and maintains high accuracy even when portions of the data are missing (Cutler et al., 2007). This may be especially beneficial when combining multiple datasets. However, due to its complex structure, interpreting Random Forests can be less intuitive and it can be difficult to determine the underlying relationships between parameters and classes. By contrast, weighted *k*‐nearest neighbor is robust to noisy data because the distance function it uses can be adjusted to accommodate large variances within the data (Zhao & Chen, 2016). However, nearest neighbor classification methods require selection of an appropriate value of the parameter *k*. Selecting a value that is too small can lead to overfitting and negative effects of noise, while selecting a value that is too large creates generalization, but reduces the negative effects of noisy data (Zhao & Chen, 2016).

Nonparametric algorithms resulted in higher classification accuracy than parametric classification methods. The success of nonparametric methods for species classification is likely a reflection of the characteristics of and the relationships among the vocalization parameters. In particular, nonparametric analyses provide more flexibility with regard to distributions, nonlinearity, parameter selection, and outliers (Friedl & Bradley, 1997; Pal & Mather, 2003; Timofeev, 2004), all of which were relevant to our dataset.

### 4.2 Parameter selection

Parameter selection played a key role in each method's ability to differentiate between species. Spectrographic software is currently limited in its ability to automatically detect and capture the full spectrum of species vocalizations (Bardeli et al., 2010; Towsey, Planitzm, Nantes, Wimmer, & Roe, 2012). Thus, manual analysis of recordings provides higher rates of accuracy, but can produce inherent error in the selection of vocalizations. Parameter selection can also be affected by the quality of recordings and underlying background noise, both of which can ultimately skew frequency and duration of measurements and limit which parameters can be included in an analysis.

Currently, there are no standard criteria for selecting parameters for analysis of avian vocalizations. For differentiation between rail species, we were limited to five frequency‐derived parameters due to high levels of pairwise correlation among initial seven parameters. The addition of new parameters describing variation in the temporal domain and aggregated phrases and notes could possibly increase the statistical power of our analyses (Thompson, LeDoux, & Moody, 1994). Although parametric classification methods require parameter selection prior to analysis, nonparametric classification methods allow for parameter selection during analysis. We conducted parameter selection prior to statistical analyses to ensure consistency of parameters across all models for comparison. Overall, the relative importance of each parameter is dependent on the classification method used.

### 4.3 Alternative processing and statistical techniques

We selected our methodology for processing and preparing the acoustic data from among many available techniques for understanding and evaluating avian vocalizations. We processed our acoustic data with a commonly used sound analysis software to facilitate transfer of knowledge to other ecologists and conservation biologist wishing to implement similar analyses. Although sound analysis software packages such as Raven Pro, Sound Analysis Pro, and AviSoft‐SASLab Pro all provide a user‐friendly interface for spectrographic analysis, they also impose constraints. Within Raven Pro, we accounted for differences in sampling rates between sites and species by adjusting the window sizes to get similar resolutions. However, we could not make the frequency resolutions exactly the same because Raven Pro only allows for discrete window size options in a pull‐down menu, thus not allowing us to enter the exact value that would make the windows equivalent. Nevertheless, the differences in the adjusted sampling rates were marginal and should not have altered the differentiation process. For parameter selection, we conducted fast Fourier transformations and selected parameters from the power spectra performed in previous studies (Bardeli et al., 2010; Towsey et al., 2012; Zollinger, Podos, Nemeth, Goller, & Brumm, 2012). Alternatively, we could have chosen to use a constant‐Q transformation to represent the spectral data (Brown & Pucketter, 1992). We did not take this approach primarily because common acoustic software packages only include the option for Fourier transformations.

An alternative technique for parameter selection is the use of Mel‐Frequency Cepstral Coefficients (MFCCs) for acoustic feature extraction. MFCCs are a signal representation method used in audio classification tasks, most frequently for human speech recognition (Davis & Mermelstein, 1980). The basis for the Mel‐frequency scale is derived from the human perceptual system, which is not the same as that of birds. Additionally, this methodology is less intuitive for practitioners to implement as it requires calculation of the MFCC parameters by segmenting calls into overlapping frames and transforming the power spectrum of each frame into logarithmic mel‐frequency spectrum using triangular filter (Davis & Mermelstein, 1980; Fagerlund 2007; Towsey et al., 2012). When using MFCCs, songs and calls are parameterized using descriptive measures derived from the temporal and spectral domains. This method has been used for automated recognition of calls of multiple avian species (Cai, Ee, Pham, Roe, & Zhang, 2007; Dufour, Artieres, Glotin, & Giraudet, 2014; Fagerlund 2007; Lee, Lee, & Huang, 2006; Potamitis, Ntalampiras, Jahn, & Riede, 2014). While MFCCs are a viable method for classifying bird songs, in certain situations they can be outperformed by other machine learning methods (Stowell & Plumbley, 2014).

Our analysis evaluated the performance of nine classification tools, but this was by no means exhaustive. Dynamic time warping has been used to match spectrograms of syllables from Indigo Buntings (*Passerina cyanea*) and Zebra Finches (*Taeniopygia guttata*) to a repository of spectrograms (Anderson, Dave, & Margoliash, 1996). Alternatively, Gaussian mixture models (GMMs) estimate the probability density function used for statistical classification by modeling complex distributions with multiple modes (Brown & Smaragdis, 2009; Kwan et al., 2006; Roch, Soldevilla, Burtenshaw, Henderson, & Hildebrand, 2007; Somervuo, Härmä, & Fagerlund, 2006). Hidden Markov models (HMMs) take GMMs a step further by modeling the temporal data in a sequence of states defined by GMMs (Clemins, 2005; Kogan & Margoliash, 1998). Using a sequence of GMMs to explain the input data, HMMs can allow for sensitivity in temporal changes within a call and can thereby be used to describe the structure of the call (Brown & Smaragdis, 2009; Chu & Blumstein, 2011; Trawicki, Johnson, & Osiejuk, 2005).

### 4.4 Intrinsic and extrinsic factors influencing call classification

Intrinsic and extrinsic factors influence the structure of vocalizations and thus the ability to distinguish between species and individuals. Marsh bird vocalizations can vary with sex, age, breeding status, and proximity to conspecifics (Conway & Gibbs, 2001; Legare, Eddleman, Buckley, & Kelly, 1999; Robertson & Olsen, 2014; Smith, 1974; Zembal & Massey, 1987). Recording artifacts can also introduce variability. The type of audio recording equipment, recording quality, distance from the bird to the recorder, and the direction the bird is calling relative to the recorder (Conway & Gibbs, 2011) are acoustic sampling variables that can be adjusted during the recording process. Environmental factors such as the strength or direction of wind, variation in temperature and humidity, level of background noise, and presence of thick vegetation can result in underlying recording artifacts that may need to be accounted for during spectrographic analysis.

The slight variation we observed in *kek* notes between King and Clapper rails may be in part a reflection of inter‐species differences in body size (Bowman, 1983; Tubaro & Mahler, 1998; Wallschager, 1980). Male Clapper rails (329.4 ± 26.7 g) are, on average, significantly smaller than male King rails (369.6 ± 34.9 g; Perkins, King, Travis, & Linscombe, 2009), although their size distributions overlap. It is therefore possible that larger bodied male King rails produce *kek* vocalizations with on average lower frequencies than those of male Clapper rails. Also, Clapper rails possessed a larger frequency range (~15% wider) than King rails. By design, our study provides a metric that allows for comparison of breeding males only, given that female King and Clapper rails are not known to *kek* (Meanley, 1969). We targeted *kek* calls since these vocalizations are heard most prominently during the breeding season, thereby providing a reliable estimate of occupancy and an opportunity to record a large sample of calls.

The potential for hybridization also presents a problem for conservation biologist and those interested in species identification through classification of vocalizations. The males of both species hide in emergent vegetation while using *kek* calls to advertise to mates (Massey & Zembal, 1987), making aural identification the primary method for species identification. However, both species are known to respond to heterospecific calls (Conway & Nadeau, 2010). Although our statistical tools were able to differentiate between the species, the introduction of hybrid individuals may alter model development and performance. Hybrid vocalizations could either have a vocalization structure more similar to one parent species or they could fall onto a gradient between species. Currently, although hybrids are known to occur, there are no published records of vocalizations produced from a confirmed hybrid individual and thus we were not able to consider such birds in our analyses. However, several statistical approaches used herein, both parametric and nonparametric, can provide an estimate of the probability of assignment to a specific classification, enabling us to identify potential hybrid individuals.

Finally, selecting the appropriate audio recording equipment is a key component to capturing vocalizations. However, understanding what equipment to choose presents challenges. Recording equipment varies by cost, durability, size, weight, sampling rate, battery life, and sound quality. The selection process becomes even more complicated when weighing budget and temporal constraints against the number of sampling locations, sampling effort, and availability of personnel. For example, while researchers with handheld shotgun microphones can adjust proximity and directionality relative to the calling bird to provide higher quality recordings, autonomous recording units (ARUs) can record for longer time periods using fewer person‐hours in the field. Ultimately, recording equipment selection depends on the goals of the project and recording quality considerations should be balanced with sampling effort.

## 5 CONCLUSION

Acoustic surveying has become an increasingly popular management tool and is the predominant surveying technique for secretive marsh birds, such as the King and Clapper rail. Our analysis aims to facilitate the process of distinguishing each species within a management area. Establishing occupancy of each species, particularly in fragile brackish coastal marshes, could be critical in determining management practices and in allocating resources for conservation. Nonparametric classification methods outperformed parametric classification methods for King and Clapper rail *kek* note differentiation. Of the nine classification tools, Random Forest was the most accurate, correctly classifying 81.1% of call notes to species, with a kappa statistic of 62.3%. This work suggests that rail vocalizations are likely difficult for human observers to tell apart, but that appropriate statistical tools may provide an alternative method for acoustic species classification where other capture‐ or genotype‐based survey techniques are not possible.

## CONFLICT OF INTEREST

None declared.

## AUTHOR CONTRIBUTION

L.L.S. and K.M.S. led initial design and collaboration of the study with assistance from all authors, L.L.S. and K.M.S. collected and spectrographically analyzed the field data, L.L.S. led statistical analysis and writing with assistance from all authors, and all authors contributed to revisions of the manuscript.

## DATA ACCESSIBILITY

Data files are available online at Dryad Digital Repository: https://doi.org/10.5061/dryad.r475hr3.
