# Supplementary material for: Quantitative acoustic differentiation of cryptic species illustrated with King and Clapper rails
Source: Ecol Evol. 2018 Nov 20;8(24):12821–31. doi: 10.1002/ece3.4711 (PMC6309001; doi:10.1002/ece3.4711)
Supplement: Supplementary file 1 [file ECE3-8-12821-s001.docx]

**SUpporting INformation**

Table S1. Descriptions of seven parameters measured for analysis of King and Clapper rail *kek* call notes (Charif et al. 2010) monitored in North Carolina and Virginia, USA, respectively, during 2015 and 2016.

| Parameter | Definition |
| --- | --- |
| Peak Frequency | Frequency at which Peak Power occurs within the selection. |
| 1st Quartile Frequency | Frequency that divides selection into 2 frequency intervals with 25% and 75% of the energy in selection. |
| 3rd Quartile Frequency | Frequency that divides selection into 2 frequency intervals with 75% and 25% of the energy in selection. |
| Inter-quartile Range Bandwidth | Difference between the 1st and 3rd quartile frequencies. |
| Frequency 5% | Frequency that divides selection into 2 frequency intervals with 5% and 95% of the energy in selection. |
| Frequency 95% | Frequency that divides selection into 2 frequency intervals with 95% and 5% of the energy in selection. |
| Bandwidth 90% | Difference between the 5% and 95% frequencies. |

Table S2. Spearman’s rank correlation coefficients for acoustic parameters measured from King and Clapper rail *kek* call notes monitored in North Carolina and Virginia, USA, respectively, during 2015 and 2016.

|  | Peak Frequency | Q1 Frequency | Q3 Frequency | IQR Bandwidth | Frequency 5% | Frequency 95% |
| --- | --- | --- | --- | --- | --- | --- |
| Q1 Frequency | 0.564 |  |  |  |  |  |
| Q3 Frequency | 0.511 | 0.450 |  |  |  |  |
| IQR Bandwidth | 0.138 | -0.249 | 0.753 |  |  |  |
| Frequency 5% | 0.254 | 0.630 | 0.135 | -0.317 |  |  |
| Frequency 95% | 0.207 | 0.184 | 0.593 | 0.508 | -0.068 |  |
| Bandwidth 90% | 0.061 | -0.130 | 0.447 | 0.581 | -0.516 | 0.890 |
